# Fruit Phenolic Profiling: A New Selection Criterion in Olive Breeding Programs

**DOI:** 10.3389/fpls.2018.00241

**Published:** 2018-02-27

**Authors:** Ana G. Pérez, Lorenzo León, Carlos Sanz, Raúl de la Rosa

**Affiliations:** ^1^Department of Biochemistry and Molecular Biology of Plant Products, Instituto de la Grasa, CSIC, Seville, Spain; ^2^Instituto Andaluz de Investigación y Formación Agraria, Pesquera, Alimentaria y de la Producción Ecológica (IFAPA), Centro Alameda del Obispo, Córdoba, Spain

**Keywords:** *Olea europaea*, olive breeding, virgin olive oil, phenolic compounds, genotype, genotype × environment interaction

## Abstract

Olive growing is mainly based on traditional varieties selected by the growers across the centuries. The few attempts so far reported to obtain new varieties by systematic breeding have been mainly focused on improving the olive adaptation to different growing systems, the productivity and the oil content. However, the improvement of oil quality has rarely been considered as selection criterion and only in the latter stages of the breeding programs. Due to their health promoting and organoleptic properties, phenolic compounds are one of the most important quality markers for Virgin olive oil (VOO) although they are not commonly used as quality traits in olive breeding programs. This is mainly due to the difficulties for evaluating oil phenolic composition in large number of samples and the limited knowledge on the genetic and environmental factors that may influence phenolic composition. In the present work, we propose a high throughput methodology to include the phenolic composition as a selection criterion in olive breeding programs. For that purpose, the phenolic profile has been determined in fruits and oils of several breeding selections and two varieties (“Picual” and “Arbequina”) used as control. The effect of three different environments, typical for olive growing in Andalusia, Southern Spain, was also evaluated. A high genetic effect was observed on both fruit and oil phenolic profile. In particular, the breeding selection UCI2-68 showed an optimum phenolic profile, which sums up to a good agronomic performance previously reported. A high correlation was found between fruit and oil total phenolic content as well as some individual phenols from the two different matrices. The environmental effect on phenolic compounds was also significant in both fruit and oil, although the low genotype × environment interaction allowed similar ranking of genotypes on the different environments. In summary, the high genotypic variance and the simplified procedure of the proposed methodology for fruit phenol evaluation seems to be convenient for breeding programs aiming at obtaining new cultivars with improved phenolic profile.

## Introduction

Virgin olive oil (VOO) is a key food within the Mediterranean diet whose daily intake has well-known benefits for human health (Estruch et al., 2013). Olive oil has traditionally been produced and consumed in the Mediterranean countries. Thus, Europe is responsible for 78% of the olive oil world production, and Spain is the largest producer with an average production of 1.3 million tons over the past 7 years, being “Picual” and “Arbequina” cultivars the two most important Spanish varieties in terms of oil production (FAOSTAT, 2016^1^). The olive possesses a substantial genetic diversity (Belaj et al., 2012) with more than 1,200 olive varieties cataloged (Bartolini et al., 2005). However, most of those cultivars are traditional as few attempts have been made to produce new varieties by systematic breeding (Bellini et al., 2002; Rallo et al., 2008; Lavee, 2013). This can be due to the limited knowledge on the real variability of olive germplasm for many of the most important agronomic and oil quality traits. Also, the long juvenile phase, high heterozygosity and scarce information on trait heritability have been important limiting factors that have negatively affected olive breeding (de la Rosa et al., 2016).

In the past few years, olive growing and olive oil production had shown an exponential increase in non-Mediterranean countries (FAOSTAT, 2016^1^). The emergence of these new olive producing areas with very different edaphoclimatic conditions respect to those of the Mediterranean countries and the necessary adaptation to intensive production systems and mechanical harvesting have driven the demand for new olive varieties. All these factors have significantly boosted the development of new and more ambitious olive breeding programs (Lavee, 2013). Thus, the objectives of most recent breeding programs are not only agronomic. In these sense, two different marketing strategies need to be fulfilled (i) producing standard quality extra-virgin olive oil at lower prices, and (ii) offering consumers a variety of extra-virgin olive oils with high quality standards and different sensory profiles. The first approach is usually related to super-intensive cultivation and highly mechanized harvesting methods, while the second is associated to preserving olive tree biodiversity and traditional methods as part of the extraordinary food traditions associated with the Mediterranean diet (Ilarioni and Proietti, 2014).

The increasing importance of the nutritional quality of olive oil for consumers and markets has led to olive-breeding programs with new nutritional targets (El Riachy et al., 2012; Rugini and De Pace, 2016). In this sense, although VOO contains a number of minor compounds with interesting biological activities, it is generally accepted that the phenolic compounds are the oil components most directly associated with its health related properties (Servili et al., 2014; Bernardini and Visioli, 2017). In addition to their nutritional properties, the phenolic compounds of VOO also have important organoleptic implications since they are the main contributors to bitter and pungent sensory descriptors. The secoiridoids compounds, containing in their molecules the phenolic alcohol tyrosol (*p*-HPEA) or its hydroxyl derivative hydroxytyrosol (3,4-DHPEA), are the most abundant class of phenolics in all olive products. Thus, the main phenolic glucosides present in the olive fruit are the secoiridods oleuropein, ligstroside and demethyloleuropein, and their hydrolytic derivatives, the dialdehydic forms of decarboxymethyloleuropein and decarboxymethylligstroside aglucones (3,4-DHPEA-EDA and *p*-HPEA-EDA, respectively) and the aldehydic forms of oleuropein and ligstroside aglucones (3,4-DHPEA-EA and *p*-HPEA-EA, respectively) are the main phenolic components in most olive oils (Montedoro et al., 2002; Pérez et al., 2014). Many studies reporting the ability of VOO phenolics to reduce chronic inflammation and oxidative damage relate these beneficial effects of VOO with the level of 3,4-DHPEA in plasma (Mateos et al., 2011; Bernardini and Visioli, 2017). This scientific evidence has led the European Union to approve a health claim on olive oil polyphenols which may be applied only for oils containing at least 250 mg/kg of hydroxytyrosol and its derivatives (European Commission, 2012).

The metabolism of phenolic compounds in the olive tree is very complex, and is modulated by genetic (Talhaoui et al., 2016) and environmental factors (Romero and Motilva, 2010) that determine the final phenolic composition of olive fruits. In a similar way, the influence of agricultural practices such as limited irrigation (Cirilli et al., 2017), optimization of pruning to increase light availability (Proietti et al., 2012) or selection of optimum harvest date (Famiani et al., 2000) have also been described. The phenolic glycosides present in the olive fruit are later transformed and modified by endogenous hydrolytic and oxidative enzymes that are activated during the oil extraction process. In this way, the phenolic profile of VOO is directly related to the phenolic content of the olive fruit (Gómez-Rico et al., 2008) and the activity of hydrolytic and oxidative enzymes during milling and malaxation (Romero-Segura et al., 2011). Any breeding program that aims to improve the phenolic composition of the VOO should consider an experimental design that allows the evaluation of all these factors. Likewise, it is important to have accurate analytical tools to find out the influence of each specific factor on the final phenolic composition of VOO. Olive breeding programs focusing on oil quality have additional limitations to those previously mentioned (de la Rosa et al., 2016) due to the very large number of genotypes but very little oil production achieved in the early stages of breeding. For this reason, it is vital to have reliable analytical methodologies to predict the composition of the oil from the analysis of the fruits. In this sense, recent studies have described significant correlations between the composition of olive fruits and oils for components such as fatty acids, sterols, tocopherols, or squalene (Velasco et al., 2014; de la Rosa et al., 2016). To the best of our knowledge, fruit phenolic profiling has not previously been used as selection criteria in olive breeding. Besides, there are no previous reports on olive comparative trials devoted to study the interaction of genetic and environmental factors that may influence fruit phenolic composition which in turn determines the phenolic profile of VOO.

The objective of this work is to describe the use of new analytical tools that allow predicting the phenolic composition of the oils from the phenolic profiling of the olive fruits, without the previous step of oil extraction, and therefore facilitating the scrutiny of large seedling populations. The predictive method developed has been used to select a new olive breeding selection producing VOO with an optimum phenolic composition.

## Materials and methods

### Plant material

The selection process on the olive breeding program of Cordoba, (Spain) is divided in three steps, seedling stage, intermediate selection and final comparative trials (León et al., 2015). In each step, the number of genotypes is reduced and the number of replications per genotype increases (de la Rosa et al., 2016). The final comparative trials are planted in different environments with a reduced number of selections according to the potential adaptability to each edaphoclimatic conditions.

In the present work, phenolic evaluation was carried out in three comparative trials planted in typical olive growing areas of Andalusia, Sothern Spain: Córdoba, Morón, and Ubeda. Ubeda has lower winter temperatures and rainfall than the other two locations, while Cordoba has the highest rainfall (Table 1). Soils mainly differ on the clay percentage (42.0% in Moron, 31.9% in Ubeda and 22.3% in Cordoba). Two breeding selections (UCI-2-68 and UCI-5-65) were planted in the selected locations together with their parents (“Picual” and “Arbequina”), following an unbalanced design. These two selections showed high productivity, oil content and oleic acid percentage in previous breeding selection stages (León et al., 2004a,b, 2007, 2011). Besides, four more breeding selections were also included in the Ubeda trial (UCI-12-85, UCI-12-104, UCI-19-79, and UCI-19-60) for having good productivity and high oil content (unpublished data). All the breeding selections come from crosses performed in 1992 to 1997, between “Picual” and “Arbequina.” All the trials have a randomized complete design with 3–4 blocks and 3–4 trees per elementary plot. Trees were planted in 2011 at 6 × 7 m distance and standard fertilization and irrigation practices were carried out. Irrigation supply (1,500 m^3^ per year and h) by in-line drippers was used to avoid water stress of plants.

**Table 1:** Mean temperatures (maximum, minimum, and average) and monthly rainfall during 2016 in the three locations studied.

	**Cordoba**	**Moron**	**Ubeda**	**Cordoba**	**Moron**	**Ubeda**	**Cordoba**	**Moron**	**Ubeda**	**Cordoba**	**Moron**	**Ubeda**
	**Avg temp (°C)**			**Max temp (°C)**			**Min temp (°C)**			**Rainfall (mm)**		
January	10.6	11.7	9.2	15.7	16.2	16.1	6.4	7.6	4.0	59.6	67.4	44.6
February	10.9	11.3	8.5	16.4	16.3	14.6	5.6	6.8	3.4	42.6	55.8	49.8
March	11.4	11.8	9.1	19.0	18.4	16.6	4.5	5.9	2.3	30.2	37.6	20.6
April	15.7	15.4	14.1	22.0	21.5	21.6	10.0	9.9	6.9	115.4	79.8	77.8
May	18.6	18.7	17.6	25.1	25.1	26.0	12.6	12.8	9.7	92.4	94.0	56.4
June	24.9	24.7	24.5	33.1	32.7	34.3	16.3	16.5	13.4	0.2	0.0	0.0
July	29.1	28.3	28.8	37.3	36.8	38.7	20.5	20.4	18.1	0.4	1.0	6.4
August	28.5	28.4	27.8	36.9	36.4	37.6	20.2	20.7	17.6	0.2	0.8	2.4
September	24.9	24.7	22.9	33.0	32.9	33.4	16.8	17.1	13.3	3.0	6.4	4.2
October	19.6	20.1	17.7	26.5	26.9	27.0	14.2	14.4	10.6	84.0	87.2	25.0
November	12.5	13.0	10.3	18.4	18.6	18.1	8.1	8.3	4.7	142.2	93.8	90.6
December	10.4	11.6	8.6	16.5	17.1	16.6	6.2	6.7	3.6	45.0	38.2	27.0
Mean temp.	18.1	18.3	16.7	25.0	24.9	25.1	11.8	12.3	9.0			
Total rainfall										615.2	562.0	404.8

Fruit samples of 2 kg were harvested per each genotype and elementary plot in 3 blocks per each of the three trials (Córdoba, Morón, and Ubeda). Breeding selections and parents were collected in a common date, in mid-November 2016, typical for olive harvesting in southern Spain, when most fruits were at turning color (de la Rosa et al., 2013). Random subsamples were taken for both direct fruit analysis and oil extraction.

### Chemicals

Reagents for extraction and other measurements were supplied by Sigma-Aldrich (St. Louis, MO). Oleuropein, verbascoside, luteolin-7glucoside, apigenin-7glucoside, rutin, apigenin, luteolin, tyrosol, hydroxytyrosol, vanillic acid, vainillin, p-coumaric acid, and ferulic acid were obtained from Sigma Chemical (St. Louis, MO, USA) and Extrasynthese (Genay, France). Non-commercial phenolic standards such as ligstroside, hydroxytyrosol-1-glucoside, or the main secoiridoids derivatives were obtained from olive leaves, fruits and oils using a high performance liquid chromatography (HPLC) preparative system.

### Olive oil extraction

Olive oil was extracted using an Abencor analyzer (Comercial Abengoa, S.A., Seville, Spain) that simulates the industrial process of VOO production on a laboratory scale (Martínez et al., 1975). Processing parameters have been precisely described in a previous study (Pérez et al., 2014).

### Extraction and analysis of fruit and VOO phenolic compounds

Fruit phenolic compounds were extracted according to a previously developed protocol (García-Rodríguez et al., 2011). Longitudinal pieces of mesocarp tissue were cut from 20 olive fruits and kept at 4°C for 24 h in DMSO (6 ml/g of fruit), containing syringic acid (24 mg/ml) as internal standard. The extracts were filtered through a 0.45 μm mesh nylon and kept at −20°C until HPLC analysis.

VOO phenolics were isolated by solid phase extraction (SPE) on a diol-bonded phase cartridge (Supelco, Bellefonte, PA) following a previously described procedure (Mateos et al., 2001). 0.5 ml of a methanol solution containing *p*-hydroxyphenyl-acetic acid and *o*-coumaric acid as internal standards was added to each oil sample (2.5 g) before the extraction.

Phenolic extracts from fruits and oils were analyzed by HPLC on a Beckman Coulter liquid chromatography system equipped with a System Gold 168 detector, a solvent module 126, an autosampler module 508 and a Waters column heater module following a previously described methodology (Pérez et al., 2014). A Superspher RP 18 column (4.6 mm i.d. × 250 mm, particle size 4 μm: Dr Maisch GmbH, Germany) at flow rate 1 mL min^−1^ and a temperature of 35°C was used for all the analyses. A total of 15 phenolic compounds were analyzed in fruit phenolic extracts: hydroxytyrosol-4-glucoside, hydroxytyrosol-1-glucoside, demethyloleuropein, verbascoside, luteolin-7-glucoside, demethylligstroside, rutin, oleuropein, comselogoside, ligstroside, luteolin 3,4-DHPEA-EA, apigenin, and *p*-HPEA-EA. The last four compounds were also analyzed in VOO extracts in which 12 other phenolic compounds were also detected: hydroxytyrosol, tyrosol, vanillic acid, vainillin, p-coumaric acid, hydroxytyrosol acetate, 3,4-DHPEA-DEA, *p*-HPEA-DEA, pinoresinol, acetoxypinoresinol, and ferulic acid. The quantification of flavones and ferulic acid was done at 335 nm while the rest of phenolic components were quantitated at 280 nm. Response factors were calculated for each phenolic compound as described previously (García-Rodríguez et al., 2011).

The tentative identification of compounds by their UV-vis spectra was confirmed by HPLC/ESI-qTOF-HRMS. The liquid chromatograph system was Dionex Ultimate 3000 RS U-HPLC liquid chromatograph system (Thermo Fisher Scientific, Waltham, MA, USA) equipped with a similar Superspher RP 18 column but with formic acid (1%) instead of phosphoric acid (0.5%) in the mobile phase. A split post-column of 0.4 mL/min was introduced directly on the mass spectrometer electrospray ion source. The HPLC/ESI-qTOF operated for mass analysis using a micrOTOF-QII High Resolution Time-of-Flight mass spectrometer (UHRTOF) with qQ-TOF geometry (Bruker Daltonics, Bremen, Germany) equipped with an electrospray ionization (ESI) interface. Mass spectra were acquired in MS fullscan mode and data were processed using TargetAnalysis 1.2 software (Bruker Daltonics, Bremen, Germany).

### Statistical analysis

Fruit samples (2 kg) were harvested per each cultivar and elementary plot in 3 blocks per each trial and random subsamples were taken for both direct fruit analysis and oil extraction. All the data were statistically evaluated using STATISTICA 5.0 (Statsoft Inc., Tulsa, OK, USA). Descriptive statistics and variability plots were obtained for the whole dataset of genotypes and environments. Correlations among phenols or group of phenols were analyzed for the whole dataset using Pearson's correlations (at *p* ≤ 0.05; *p* ≤ 0.01; *p* ≤ 0.001). A subset of data (4 genotypes and 3 environments) was used to evaluate the relative contribution of genetic and environmental factors on the phenolic variability by means of ANOVA and separation of the means was obtained at *p* ≤ 0.05 by least significance differences (LSD). Principal component analysis (PCA) was used to evaluate the levels of association among the phenolic compounds from the cultivars and advanced breeding selections under study. PCA was applied to the same subset of data used for ANOVA (4 genotypes and 3 environments) and to a second subset of data containing all the genotypes in a single environment.

## Results

### Identification and quantitation of the main phenolic components of olive fruits and oils from new advanced breeding selections

The analysis of the fruit phenolic extracts allowed identifying a great variability in the profile of phenolic glycosides of the eight olive genotypes and three environments under study. Demethyloleuropein, was the most abundant phenolic compound with a mean content of 8,787.4 μg/ g fruit and a range of variability from 26.1 to 23,937.9 μg/ g fruit. This was followed by oleuropein with a significantly lower mean value (4,966.2 μg/ g) and a lower range (175.2–16,542.2 μg/g). The mean contents of verbascoside, ligstroside and luteolin-7-glucoside were considerably lower than those of demethyloleuropein and oleuropein: 825.2, 497.2, and 391.06 μg/g respectively.

Similarly, a great variability was also observed in terms of phenolic components of the oils. Tyrosol (p-HPEA) and hydroxytyrosol (3,4-DHPEA) derived compounds were the most important class of phenolic compounds found in all VOOs. Among them 3,4-DHPEA-EDA was the most abundant phenolic component in the studied oils with a mean content of 234.1 μg/g of oil in the range 27.1–576.81 μg/g. The tyrosol derivative p-HPEA-EDA was the second most abundant component (mean value of 148.2 μg/g and range 23.3–444.11 μg/g) followed by another hydroxytyrosol derivative 3,4 DHPEA-EA (mean value 85.3 and range 14.2–410.1 μg/g) while lower contents were found for p-HPEA-EA (mean value 15.87 μg/g) and 3,4-DHPEA-acetate (mean value 11.06 μg/g). Significant amounts of lignans (acetoxypinoresinol and pinoresinol), flavones (luteolin and apigenin), and phenolic acids (cinnamic acid, p-coumaric acid, vanillic acid, and ferulic acid) were also found in the oils obtained from the eight genotypes and three environments analyzed. The highest variability ranges found among them, although less important from a quantitative point of view than those previously mentioned for tyrosol derived compounds, were those of acetoxypinoresinol which possesses promising anticancer activity (mean value 23.7 and range 10.2–74.33,4 μg/g) (Menéndez et al., 2008) and luteolin (mean value 7.32 μg/g).

### Genetic and environmental effects on the phenolic composition of olive fruit and oil

**Figure 1:**
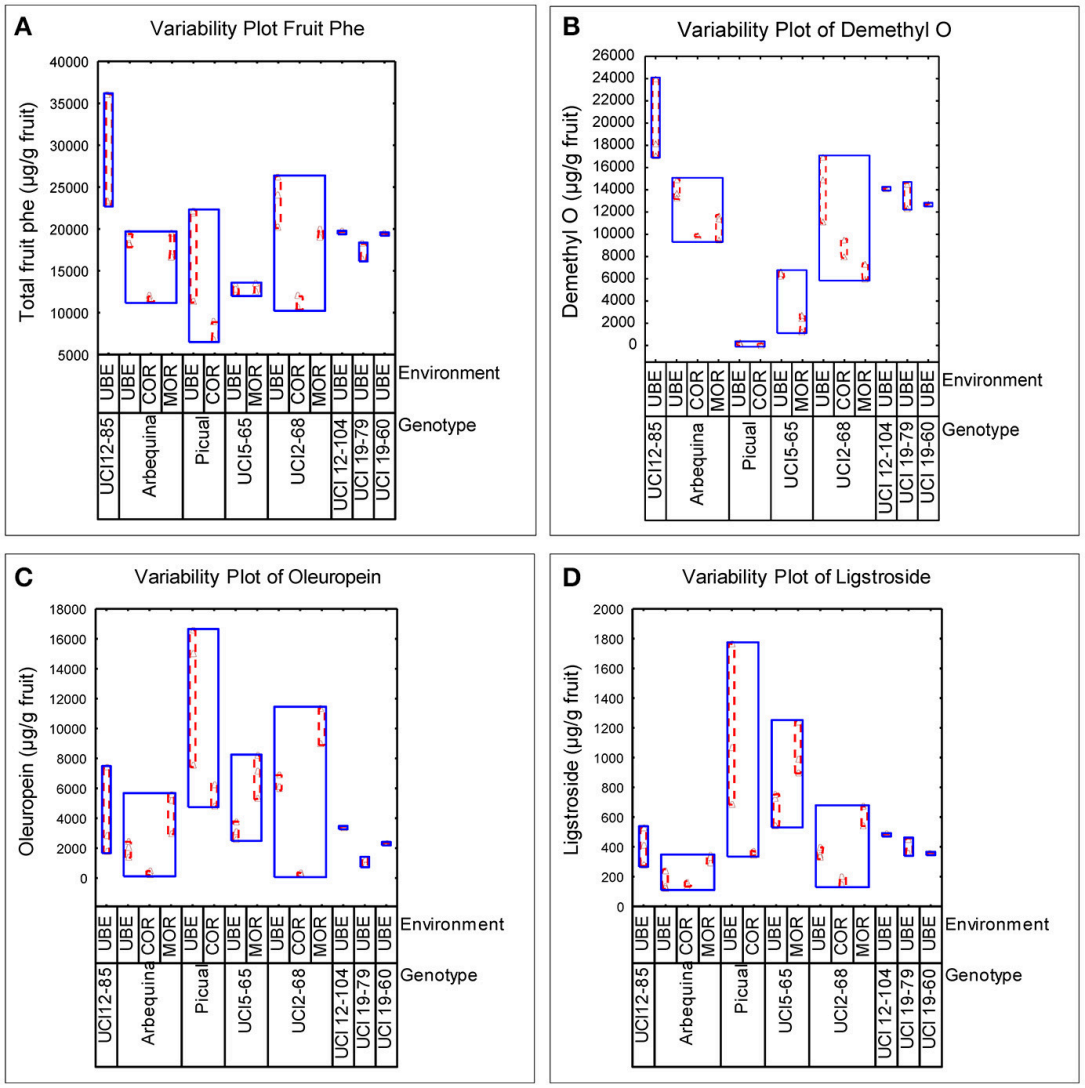
Variability plots of the main phenolic compounds (μg/g fruit pulp) analyzed in fruits from different genotypes and environments. **(A)** Total fruit phenolics. **(B)** Demethyloleuropein. **(C)** Oleuropein. **(D)** Ligstroside.

**Figure 2:**
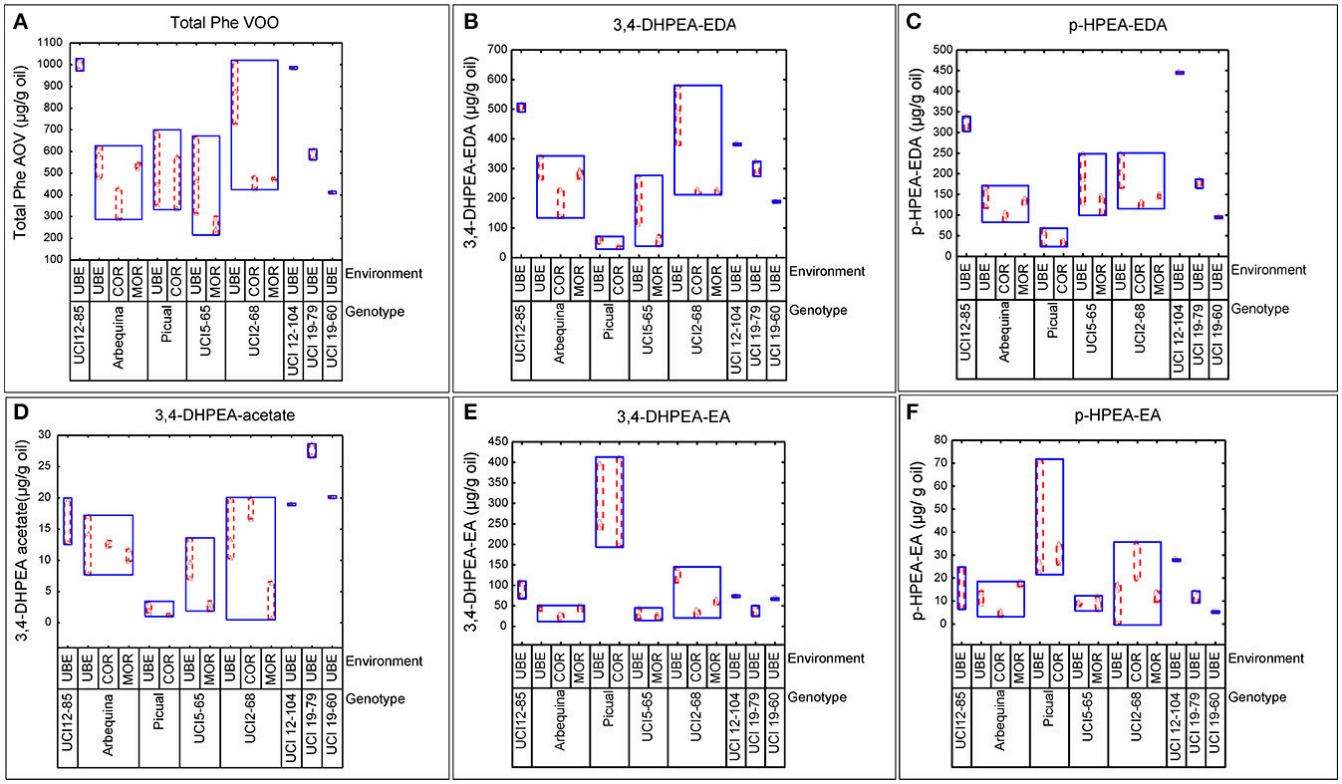
Variability plots of the main phenolic compounds (μg/g oil) analyzed in oils from different genotypes and environments. **(A)** Total VOO phenolics. **(B)** 3,4-DHPEA-EDA. **(C)** p-HPEA-EA. **(D)** 3,4 DHPEA-acetate. **(E)** 3,4-DHPEA-EA. **(F)** p-HPEA-EA.

The well-known influence of edaphoclimatic parameters on the quality of VOO makes extremely important to include different environments in the evaluation of advanced olive breeding selections (León et al., 2016). In the present work, the phenolic evaluation was carried out in three comparative trials (Morón, Córdoba, and Ubeda), representing different edaphoclimatic areas in Southern Spain. The variability specifically induced by both factors, genotype and environment, on the phenolic profile of fruits and oils was analyzed. Figure 1, graphically shows the variability observed in terms of the total phenolic content in the fruits, and in the content of the three main secoiridoid glycosides. Thus, among the genotypes grown in different environments, the highest phenolic content was always found in fruits grown in the environment Ubeda, while the lowest content was generally associated to fruits from Córdoba. However, the range of this environmental variability was different for each genotype. Figure 2 graphically shows the variability plots of the main phenolic components found in VOO. The influence of the environmental factor on the oils was in good agreement with that observed in the fruits. As previously reported for fruit phenolic, in those genotypes grown in the three different environments, higher phenolic contents were always associated to oils from the environment Ubeda. The highest phenolic contents were found in oils obtained from breeding selections UCI 12-85 and UCI 12-104 grown in Ubeda. Besides their extremely high phenolic content, these two genotypes also possess very high levels of p-HPEA-EDA (320 and 450 μg/g oil, respectively), which is closely related to the pungency of VOO (Andrewes et al., 2003).

**Figure 3:**
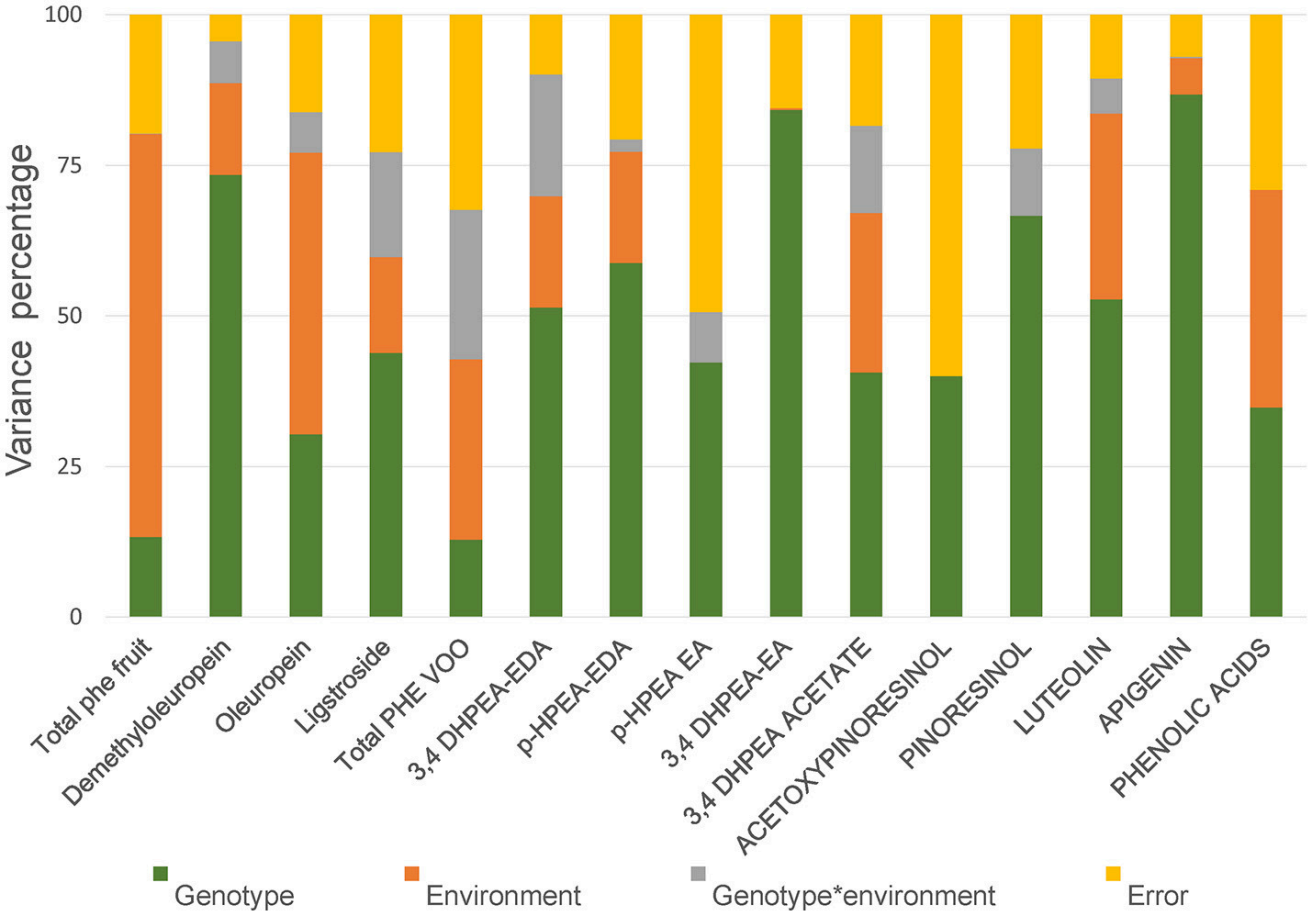
Variance percentage attributed to genotype, environment, and genotype * environment.

In order to evaluate the relative contribution of genetic and environmental factors on the variability observed in the phenolic content of fruits and oils a subset of data from the genotypes “Arbequina,” “Picual,” UCI 5-65, and UCI 2-68, was subjected to analysis of variance. Minor phenolic components were excluded and only 15 phenolic variables were included in the analysis. Four variables were selected in fruits (total phenolics; demethyloleuropein, oleuropein and ligstroside) and 11 variables were selected in VOOs (total phenolics; 3,4 DHPEA-EDA; p-HPAE-EDA; 3,4 DHPEA-EA; HPEA-EA, 3,4 DHPEA-acetate, pinoresinol, acetoxypinoresinol, luteolin, apigenin, and the sum of the main phenolic acids (cinnamic acid, p-coumaric acid, vanillic acid, and ferulic acid). The influence of each factor was estimated by the percent of the total variance (Figure 3). Analysis of variance shows a significant effect (*p* ≤ 0.05) of genotype for all the phenolic compounds analyzed in fruits and VOOs. The environment was the major contributor to the variance of the total phenolic content of the fruits (66.9%), fruit secoiridoids (59.7%), oleuropein content (46.8%), and to a lesser extent to the variance of the total phenolic content of the oils (29.99%). On the contrary, the genotype × environment effect was only significant (*p* ≤ 0.05) for demethyloleuropein and 3,4 DHPEA-EDA. To analyze this information in detail, the mean values of the 15 selected variables in the four genotypes and three environments analyzed were compared (Table 2). Significant differences were found for key phenolic components among the four genotypes. UCI 2-68 displayed the highest phenolic content in both, fruit and VOO. The content of demethyloleuropein, the most abundant phenolic glycoside found in the olive fruits analyzed was significantly higher in UCI 2-68 and “Arbequina,” with moderate levels found in UCI 5-65 and very low amount detected in “Picual” fruits. The genotype UCI 2-68 also had the highest contents of 3,4 DHPEA-EDA, p-HPEA-EDA, 3,4 DHPEA-acetate, and pinoresinol.

**Table 2:** Phenolic components (μg g^−1^) of fruits (lowercase) and oils (uppercase letters) with regards to genotype and environment.

	**Total phe fruit**	**Demethyloleuropein**	**Oleuropein**	**Ligstroside**	**Total PHE VOO**	**3,4 DHPEA-EDA**	**p-HPEA-EDA**	**p-HPEA EA**	**3,4 DHPEA-EA**	**3,4 DHPEA ACETATE**	**ACETOXYPINORESINOL**	**PINORESINOL**	**LUTEOLIN**	**APIGENIN**	**PHENOLIC ACIDS**
**GENOTYPES**															
Arbequina	16387.3 ab	11771.5 a	2537.4 c	220.4 b	502.3 b	265.4 b	128.2 b	12.0 b	37.7 b	12.0 a	29.1 ab	2.6 c	8.2 b	2.9 b	1.4 b
Picual	141114 bc	132.6 c	10029.0 a	847.3 a	482.2 b	49.7d	42.7 c	36.1 a	298.5 a	1.9 b	38.7 a	3.8 b	6.1 c	2.0 c	2.0 a
UCI2-68	18800.5 a	10527.0 a	5676.7 b	373.7 b	640.6 a	333.6 a	166.0 a	15.7 b	77.9 b	12.5 a	19.6 b	4.5 a	3.9 d	0.8 d	1.3 b
UCI5-65	12706.0 c	4300.5 b	4998.5 b	858.2 a	368.4 b	121.2 c	156.6 ab	9.2 b	25.5 b	6.4 b	15.9 b	2.7 c	11.1 a	5.3 a	1.1 b
**ENVIRONMENTS**															
cordoba	10170.5 b	6228.9 b	2050.1 b	225.1 b	423.9 b	146.2 b	84.2 b	20.7	118.6 a	10.7 a	30.4	3.9 a	3.2 b	1.2 b	1.1 b
Moron	16220.9 a	6534.3 b	6830.4 a	663.2 a	414.9 b	181.2 b	133.3 a	13.0	39.2 b	5.8 b	20.5	2.9 b	7.9 a	3.5 a	1.5 a
Ubeda	18225.4 a	8723.2 a	6102.4 a	592.3 a	605.1 a	258.9 a	147.4 a	17.8	122.3 a	10.0 a	26.1	3.3 b	8.9 a	3.0 a	1.5 a

*Different letters indicate LSD between genotypes and environments ANOVA (P ≤ 0.05)*.

### Relationship between fruit and VOO phenolic components

To investigate the possibility to predict the phenolic composition of the VOO from the analysis of the phenolic profiles of the fruits Pearson‘s correlation coefficients were computed using the data obtained from all the fruits and oils analyzed in this study (Table 3). Significant positive correlation was found between total fruit phenolics and total VOO phenolic contents (*r* = 0.685) and even a slightly higher coefficient was calculated for fruit secoiridoids compounds and the total phenolic content of the oil (*r* = 0.735). The highest correlation found between individual fruit phenolic compounds and VOO phenolic content was found for demethyloleuropein (*r* = 0.650). Curiously, oleuropein content correlated poorly to total fruit phenolics (*r* = 0.289) and no correlation at all was found between oleuropein content and the total phenolic content of the oil (*r* = −0.057). Data shown in Table 3 also provides relevant information on the relationship between the different phenolic components of VOO. Thus, 3,4-DHPEA-EDA was highly correlated to total AOV phenolics (*r* = 0.850) followed by p-HPEA-EDA (*r* = 0.759) while significantly lower correlation coefficients were found for the secoiridoids with monoaldehyde structure, 3,4-DHPEA-EA and p-HPEA-EA (*r* = 0.217 and *r* = 0.144, respectively).

**Table 3:** Pearson's correlation coefficients among the main phenolic compounds found in fruits (lowercase letters) and oils (uppercase letters) from all genotypes and environments.

	**Total phe fruit**	**Fruit secoiridoids**	**Demethyl O**	**Oleuropein**	**Ligstroside**	**Verbascoside**	**TOTAL PHE VOO**	**AOV SECOIRIDOIDS**	**3,4-DHPEA**	**p-HPEA**	**3,4-DHPEA AC**	**3,4-DHPEA-EDA**	**p-HPEA-EDA**	**ACETOXY-P**	**PINORESINOL**	**3,4-DHPEA-EA**	**p-HPEA-EA**	**CINNAMIC ACID**	**LUTEOLIN**	**APIGENIN**
Total phe fruit	1																			
Fruit secoiridoids	^***^0.991	1																		
Demethyl O	^***^0.594	^***^0.643	1																	
Oleuropein	0.289	0.241	^**^−0.584	1																
Ligstroside	0.046	0.013	^**^−0.644	^***^0.765	1															
Verbascoside	0.092	−0.027	−0.318	^*^0.397	0.327	1														
TOTAL PHE VOO	^***^0.685	^***^0.735	^***^0.650	−0.057	−0.197	−0.291	1													
VOO SECOIRIDOIDS	^***^0.688	^***^0.740	^***^0.653	−0.071	−0.149	−0.244	^***^0.999	1												
3.4-DHPEA	−0.079	−0.120	−0.584	^**^0.585	^***^0.586	0.310	−0.232	−0.082	1											
p-HPEA	0.102	0.082	−0.234	0.304	^**^0.501	0.122	0.129	0.272	^***^0.674	1										
3.4-DHPEA AC	0.261	0.288	^***^0.758	^***^−0.668	^**^−0.532	−0.256	0.413	0.406	^**^−0.565	−0.206	1									
3.4-DHPEA-EDA	^***^0.695	^***^0.753	^***^0.900	−0.343	^*^−0.492	^*^−0.388	^***^0.850	^***^0.858	−0.421	0.077	^***^0.612	1								
p-HPEA-EDA	^***^0.478	^***^0.534	^***^0.663	−0.298	−0.175	^*^−0.479	^***^0.759	^***^0.770	−0.183	0.307	^**^0.477	^***^0.756	1							
ACETOXY-P	0.026	0.020	−0.235	0.335	0.234	0.215	0.122	0.089	−0.033	−0.016	−0.258	−0.155	−0.317	1						
PINORESINOL	^**^0.361	^**^0.355	^**^0.347	−0.103	−0.121	−0.154	^***^0.630	^***^0.638	0.027	^**^0.381	0.279	^**^0.441	^***^0.694	−0.095	1					
3.4-DHPEA-EA	0.060	0.037	^*^−0.469	^***^0.611	^*^0.387	^*^0.361	0.217	0.203	0.280	0.198	^*^−0.437	−0.213	−0.343	^***^0.669	0.117	1				
p-HPEA-EA	−0.046	−0.056	^*^−0.388	^**^0.480	^**^0.435	0.179	0.144	0.124	0.155	0.142	−0.257	−0.249	−0.157	^***^0.698	0.126	^***^0.721	1			
CINNAMIC ACID	^*^0.371	0.326	−0.125	^***^0.609	^*^0.357	^**^0.492	0.161	0.153	0.321	0.261	−0.215	−0.007	−0.213	0.301	−0.009	^***^0.557	0.316	1		
LUTEOLIN	−0.003	−0.038	0.045	−0.096	0.163	−0.083	−0.022	−0.033	−0.018	0.042	0.093	0.033	0.112	−0.229	^*^0.398	−0.201	0.265	0.028	1	
APIGENIN	−0.230	−0.272	−0.225	−0.008	^*^0.362	0.028	−0.280	−0.288	0.161	0.188	−0.256	−0.259	−0.034	−0.163	^*^0.379	−0.257	0.257	0.035	^**^0.776	1

Marked correlations are significant at

* P ≤ 0.05,

** P ≤ 0.01,

*** *P ≤ 0.001*.

### Phenolic profiling as a selection criterion in olive breeding

**Figure 4:**
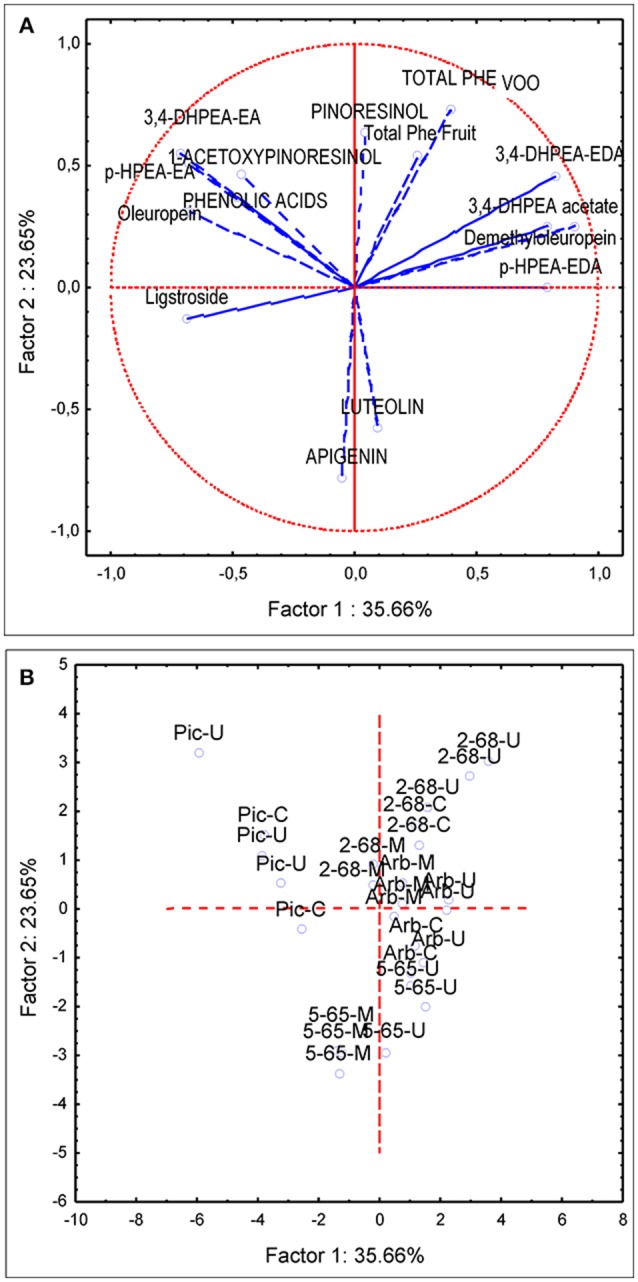
Principal component analysis of the main phenolic components of fruits (lowercase) and oils (uppercase letters) from olive genotypes: Arbequina, Picual, UCI 5-65, and UCI 2-68 grown in three environments. **(A)** vector distribution of the phenolic compounds. **(B)** distribution of genotypes-environments.

**Figure 5:**
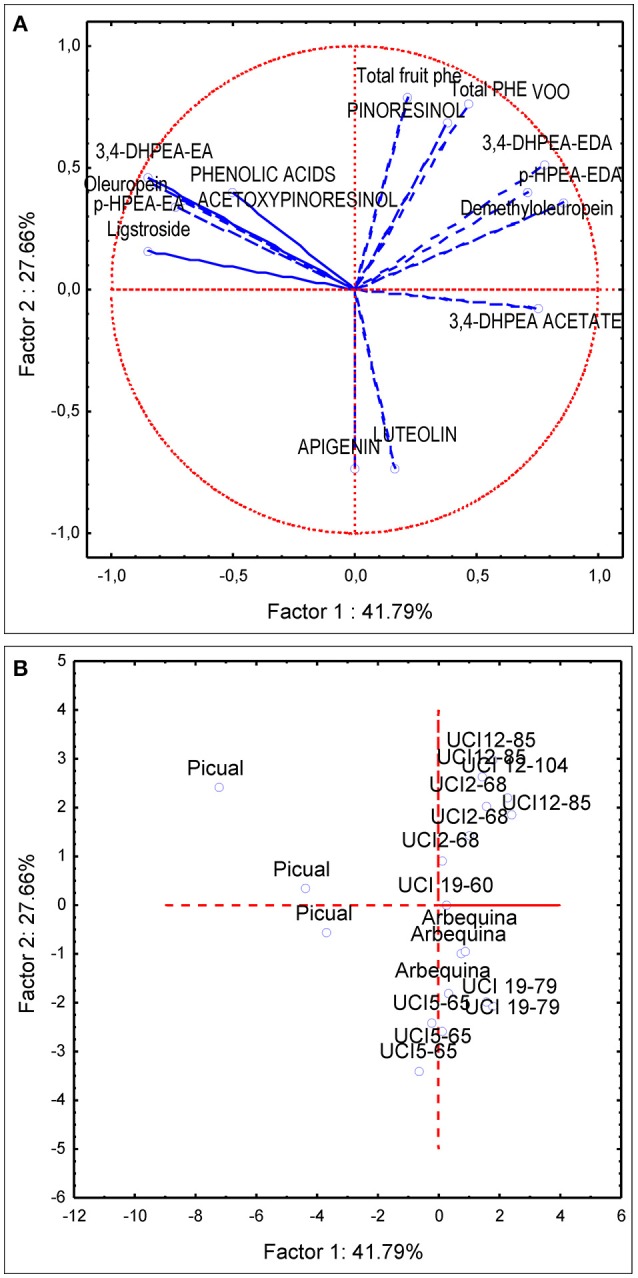
Principal component analysis of the main phenolic components of fruits (lowercase) and oils (uppercase letters) from olive genotypes grown in Ubeda. **(A)** vector distribution of the phenolic compounds. **(B)** distribution of genotypes.

PCA analysis was first applied to the same subset of data used for the variance analysis (four genotypes and three environments). The first and second principal components described 60% of the total variability (PC1 35.66 and PC2 23.65%). PC1 was strongly linked to demethyloleuropein (*r* = 0.90), 3,4-DHPEA-EDA (*r* = 0.82), p-HPEA-EDA (*r* = 0.78) while negatively correlated to oleuropein (*r* = −0.67), 3,4-DHPEA-EA (*r* = −0.71), p-HPEA-EA (*r* = −0.67). PC2 was positively correlated to total VOO phenolic content (*r* = 0.72), pinoresinol (*r* = 0.633) and to a lesser extent to acetoxypinoresinol (*r* = 0.46) and negatively correlated to luteolin and apigenin content (*r* = −0.57 and *r* = −0.78, respectively). The PCA bi-plot (Figure 4) shows the contribution of the PCA analysis to sample profiling for genotype and environment. The distribution of phenolic profiles in the PCA bi-plot suggests a greater influence of genotype vs. environment. In this sense, the distinction of different environments within each genotype is not evident in the four genotypes studied. Thus, the profiles of UCI 5-65 and UCI 2-68 grown in different environments were only partially segregated in the scatter-plot. On the contrary, when PCA analysis was applied to all the breeding selections grown in the environment Ubeda the eight genotypes were clearly separated in the scatter plot (Figure 5).

## Discussion

### Phenolic composition of new advanced olive breeding selections. influence of genetic and environmental factors

The major aim of the olive breeding program of Cordoba is to select new olive cultivars that, together with their good agronomic characteristics, are able to produce oils with an optimum nutritional and organoleptic quality. The genotypes included in this study are advanced selections from this program which in previous breeding selection stages have shown good productivity and high oil content (León et al., 2004a,b, 2007, 2011). The phenolic profiles of fruits and oils of these new breeding selections together with their parents (“Picual” and “Arbequina”) showed a great variability in the three environments studied (Figures 1, 2). The greatest variability in the phenolic profile of the fruits was associated to demethyloleuropein, oleuropein and verbascoside. The mean value found for demethyloleuropein (8787.4 μg/g) is lower than the mean content analyzed in the “Arbequina” fruits grown in the three environments selected in this study shown in Table 1 (11,771.5 μg/g) but quite similar to other values recently reported for “Arbequina” fruits also grown in south Spain (Romero et al., 2017). However, the significant content of demethyloleuropein found in all the breeding selections analyzed it is quite remarkable considering the fact that this phenolic glycoside is only present in a very short number of traditional olive cultivars (Gómez-Rico et al., 2008). The mean content of oleuropein found among the advanced breeding selections (Figure 1) was lower than the mean value found for “Picual” fruits in the environments selected in this study (Table 2) and also lower than those recently reported for Picual fruits grown in seven orchards representatives of southeast Spain (Romero et al., 2017). The high content of verbascoside found in the breeding selections analyzed might also be of interest from a nutritional point of view given the biological properties and clinical potential described for this compound (Alipieva et al., 2014). However, the content of this glucoside is not so relevant in relation to VOO phenolic composition since verbascoside, due to its chemical structure, is not hydrolysable by olive β-glucosidase so that no significant hydrolytic derivatives of this glucoside are found in VOO (Romero-Segura et al., 2012). The two most relevant phenolic compounds found in the oils of the eight genotypes were 3,4 DHPEA-EDA and p-HPEA-EDA. The variability range of 3,4 DHPEA-EDA was quite similar to that previously analyzed in 136 olive seedlings from a single cross Picual × Arbequina (Pérez et al., 2014) and significantly higher than those found among the crosses “Arbequina” × “Arbosana” and “Sikitita” × “Arbosana” (El Riachy et al., 2012). The well-known biological activity of 3,4 DHPEA-EDA (Grasso et al., 2007; Bernardini and Visioli, 2017) and its high content suggest a key contribution of this compound to the antioxidant activity of the oils. Similarly, the high content of p-HPEA-EDA found among the analyzed breeding selections may also have important quality implications, both nutritional and organoleptic, due to its relation to oil pungency and to its anti-inflammatory properties (Lucas et al., 2011).

One of the main goals of this study was to estimate the relative influence of genetic and environmental factors on the phenolic composition of olive breeding selections. The variability plots obtained for the main phenolic components of the fruits and oils (Figures 1, 2) and the comparison of means (Table 2) show the specific contribution of environments and genotypes to the phenolic variability found in this study. Rainfall and/or irrigation regime of the olive tree it is probably the most studied environmental factor influencing the composition of VOOs, (Romero and Motilva, 2010). In this sense, given that the water applied by irrigation was similar in the three environments, the major differences in water availability corresponds to rainfall (Table 1), that was higher in Cordoba respect to the other two locations, with the lowest value associated to Ubeda. The overall higher phenolic content found in fruits and oils from Ubeda could be related with the lower rainfall of this location and similarly, the lower phenolic content of genotypes grown in Cordoba could be explained by its highest rainfall. The increase of phenolic content in VOO with reduced water availability has been well documented for olive (Marra et al., 2016; Cirilli et al., 2017) although this relation has not always been clearly observed (Pierantozzi et al., 2014). However, taking into account that the three environments differs on many aspects (soil type, temperature regime, rainfall, etc.), differences on phenolic composition among the three environments could not be directly attributed to a single environmental factor. In relation to the contribution of the genotype, the variability pattern of UCI 2-68 in total VOO phenolics is in good agreement with that observed for demethyloleuropein content (Figure 1B), which is the precursor of 3,4-DHPEA-EDA the most abundant phenolic compound analyzed in the oils of UCI 2-68, (Figure 2B). Similarly, the highest variability of “Picual” oils for the 3,4 DHPEA-EA and p-HPAE-EA contents (Figures 2E,F) correlates with that previously mentioned for oleuropein and ligstroside contents in the same fruits (Figures 1C,D). The major contribution of the genotype to the phenolic variability was clearly demonstrated after calculating the percent of the total variance (Figure 3). Thus, while a significant effect of the genotype was found for all the phenolic compounds, the environment was only a major contributor to the variance of the total phenolic content of the fruits (66.9%). According to variance components, the strongest genotypic effects were observed in the contents of apigenin (86.8%), 3,4-DHPEA-EA (84.2%), demethyloleuropein (73.4%), and pinoresinol (66.6%). As shown in Figure 3, the genotype × environment effect was only significant (*p* ≤ 0.05) for demethyloleuropein and 3,4 DHPEA-EDA, although in both cases the relative contribution to total variance is very low compared to the main effects.

The comparison of the mean values of the 15 selected variables in the four genotypes and three environments (Table 2) showed statistically significant differences among the phenolic profiles. The oils from selection UCI2-68 had the highest phenolic content in fruits and oils, and the highest levels of 3,4-DHPEA-EDA. In contrast to previous literature on olive oil, the mean phenolic contents analyzed in the oils from “Picual” and “Arbequina” cultivars (482,2 and 502 μg/g, respectively) were not significantly different. In this sense, some studies have also reported the similarities between the phenolic contents of VOOs obtained from fruits from these two cultivars harvested at specific ripening stages (García González et al., 2010; Pérez et al., 2014). The comparison of the mean values determined in each environment indicates that, with the exception of 3,4-DHPEA-EDA and Pinoresinol, significantly lowest phenolic contents were always found in the fruits and oils from Cordoba. On the contrary, although the values found in fruits and oils from Ubeda were usually higher than in the other two environments, for most traits the differences between Ubeda and Moron were not statistically different.

According to the data obtained, the phenolic profile seems to be dependent on the genotype with only quantitative, but no-qualitative, differences associated to the environmental factor. Although it is clear that variations among years might be added to that observed between environments, considering that the climatic conditions (maximum, minimum, and average temperature and rainfall) of 2016 were in the average range of the last 10 years, the results could be extendable to other seasons.

### Relationship between fruit and VOO phenolic components

One of the main objectives of this study was to investigate the suitability of fruit phenolic profiling to predict the phenolic composition of VOO from olive breeding selections. The hydrolysis of secoiridoid glycosides seems to be the key step in the biosynthesis of phenolic components during the extraction of VOO (Obied et al., 2008). In previous studies we have demonstrated that during the milling of the olive fruits, cell integrity is disrupted and phenolic glycosides are transformed to their corresponding aglycones (3,4-DHPEA-EDA, p-HPEA-EDA, 3,4-DHPEA-EA, p-HPEA-EA, luteolin or apigenin) by a highly specific olive β-glucosidase (Romero-Segura et al., 2012). The secoiridoid derivatives formed may be further hydrolyzed to simple phenolic compounds such as 3,4 DHPEA or p-HPEA. Other compounds such as the lignans, pinoresinol and 1-acetoxypinoresinol, not detected in the olive pulp, are presumably formed in the olive seed and only liberated and transferred to the oil after olive stone crushing (Klen et al., 2015). However, a number of studies have reported that only a minimal amount of the phenolic compounds formed during the milling of the olive fruits and the subsequent malaxation of olive pastes are transferred to the oil. This transfer being cultivar dependent (Talhaoui et al., 2016) and significantly different for each class of compounds, with the highest transfer rate corresponding to secoiridoids compounds, followed by flavonoids and simple phenols. The Pearson‘s correlation coefficients computed with all the phenolic components analyzed in fruits and oils could provide useful information on this issue (Table 3). The correlation between the total phenolic content of fruits and oils was (*r* = 0.685) and slightly higher between total secoiridoids from fruits and oils (*r* = 0.740). Higher correlations have been reported for fatty acids (*r* = 0.98), tocopherols (*r* = 0.96), and other compounds analyzed in olive fruit and VOO (Velasco et al., 2014). However, those compounds are already present in the olive fruit tissue while the biosynthesis of oil phenolic components may involve a number of complex biochemical reactions (Obied et al., 2008; Klen et al., 2015) which are also affected by the oxidative degradation catalyzed by olive polyphenol oxidase and peroxidase (García-Rodríguez et al., 2011). The significant positive correlation found between demethyloleuropein and the VOO phenolic content (*r* = 0.650) contrasts with the low value found for oleuropein (*r* = −0.057). However, the very different correlation coefficients found for both secoiridoid glycosides are in good concordance with the higher mean value found for demethyloleuropein compared to that of oleuropein in the fruits of the eight genotypes analyzed (Figure 1). Similarly, the highest Pearson‘s coefficients between individual fruit and oil phenolic components were obtained for demethyloleuropein and 3,4-DHPEA-EDA (*r* = 0.900), which in turn is the most abundant secoiridoid compound in the oils analyzed in this study (Figure 2). This data also corroborates previous findings on the high specificity of the olive β-glucosidase which forms 3,4-DHPEA-EDA as the unique product of demethyloleuropein hydrolysis while 3,4-DHPEA-EA is the main, but not the unique product found after oleuropein hydrolysis (Romero-Segura et al., 2012). In this sense, the correlation coefficient found for 3,4-DHPEA-EA and oleuropein was significantly lower (*r* = 0.611) than that mentioned for 3,4-DHPEA-EDA and demethyloleuropein. Positive correlation coefficients were also found between demethyloleuropein and p-HPEA-EDA (*r* = 0.663) and demethyloleuropein and 3,4-DHPEA acetate (*r* = 0.758) although no conclusive data has been obtained so far on their biosynthesis. In a similar way, ligstroside content positively correlated to p-HPEA-EA (*r* = 0.435) which suggests a similar biosynthetic pathway for the latter compound to that described for 3,4-DHPEA-EA (Romero-Segura et al., 2012). The also high correlation coefficient found between ligstroside and oleuropein (*r* = 0.765) points to a common biosynthesis for both glycosides not fully demonstrated yet (Obied et al., 2008).

Data shown in Table 3 also reveal significant differences between the major phenolic components of the VOO, with very high correlation values between 3,4-DHPEA-EDA (*r* = 0.850) and p-HPEA-EDA (*r* = 0.759) and the total phenolic content of the oils but very low coefficients for 3,4-DHPEA-EA and p-HPEA-EA. Similar correlations were reported by El Riachy et al. (2012) for these monoaldehydic compounds in the analysis of two different segregating populations although non-significant correlation was found for the major secoiridoid component of VOO, 3,4-DHPEA-EDA. An interesting relation was also found also between 3,4-DHPEA-EDA and the non-secoiridoid 3,4-DHPEA acetate (*r* = 0.612). This correlation value, and that previously mentioned between demethyloeluropein and 3,4-DHPEA-acetate, seem to support the formation of 3,4-DHPEA-acetate not by simple hydroxytyrosol acetylation but through a more complex biochemical pathway involving the cleavage of the aglycone formed after demethyloleuropein deglucosilation. The elucidation of 3,4-DHPEA-acetate biosynthesis could have great interest from a biotechnological point of view given the enhanced bioavailability of this hydroxytyrosol derivative (Mateos et al., 2011). Pinoresinol was the non-secoiridoid compound which best correlated to AOV phenolic content (0.630) while a very low correlation coefficient was calculated for acetoxypinoresinol (*r* = 0.122).

Among all the information obtained from the Pearson correlation coefficients computed, it is important to emphasize that the significant correlations found between specific phenolic compounds, or groups of phenolic compounds, in the olive fruit, and the phenolic content of AOV may have an important predictive value.

### Phenolic profiling as a selection criterion in olive breeding

The first and second principal components from the PCA applied to the data set used for analysis of variance described 60% of the total variability found in the four genotypes and three environments (Figure 4). This value is quite similar to that found in previous studies on the phenolic variability of olive progenies (El Riachy et al., 2012; Pérez et al., 2014). The distribution of phenolic profiles in the PCA bi-plot seems to confirm the conclusion raised after the analysis of variance, since most of the variability shown in Figure 4 corresponds to genotype rather than to environment. In this sense, while the four genotypes are clearly segregated, the distinction among the different environments within each genotype is not always possible. Thus, the profiles of UCI 5-65 and UCI 2-68 grown in different environments were only partially segregated in the scatter-plot.

The genotype Picual, located in the second quadrant of the plot (Figure 4B), is clearly separated from the other three genotypes. According to the vector distribution (Figure 4A) oleuropein, 3,4 DHPEA-EA, p-HPEA-EA, and acetoxypinoresinol are related, and located in the second quadrant in which the genotype Picual is also included (Figure 4B). The other three genotypes are mainly located in the right part of the scatter plot, mostly in the first and fourth quadrants. According to the scatter plot the phenolic profiles of the two analyzed breeding selections (UCI 5-65 and UCI 2-68) seem to be closer to “Arbequina” than to “Picual” cultivar. In this sense, UCI 2-68, located in the first quadrant has a phenolic composition quite similar to that of “Arbequina” cultivar but a higher phenolic content. On the contrary, selection UCI 5-65 exhibits also a similar phenolic pattern, with higher luteolin and apigenin levels that increase its potential antioxidant properties (Rice-Evans et al., 1995), but with a significantly lower total phenolic content. As shown in Figure 4B, the overall higher phenolic content of fruits and oils from Ubeda are confirmed by the upper position of 5-65-U and 2-68-U in their respective groups. On the contrary, the influence of environmental factors seems to be less important in “Arbequina,” located in the central area of the plot and in “Picual” cultivar located along the second quadrant.

The effect of environmental factors on the phenolic profile of new breeding selections may provide very relevant information within an olive breeding program. In this sense, the total phenolic content of oils from UCI 5-65 grown in Ubeda is 482,2 μg/g oil, but this content is significantly lower in other environments (Figure 2A). Thus, the very low phenolic content found in the oils obtained from fruits grown in Moron (253.0 μg/g oil) could negatively affect the nutritional quality of these oils and could exclude them from the European health claim (European Commission, 2012). Taking into account the minimum values established by the EFSA for VOO phenolic health claim (250 μg/g oil), and the additional benefits of a medium-high phenolic content for the organoleptic properties and the stability of VOO, the breeding selection UCI 2-68 is clearly superior to UCI 5-65 in terms of phenolic composition.

When PCA analysis was applied to breeding selections grown in the same environment genotypes are clearly segregated (Figure 5). The selection UCI 12-85, with the highest oil phenolic content (999 μg/g oil) and the selection UCI 12-104 are located in the upper part of the first quadrant, above the selection UCI 2-68. The high phenolic content of the breeding selection UCI 12-85 may have potential in terms of nutritional quality. However, it is important to point out that the very high levels of secoiridoid compounds in its oil may also have a negative impact in its organoleptic properties. In this sense, the VOO from this selection has the highest contents of p-HPEA-EDA (321 μg/g oil) which is highly related to the pungency of VOO (Andrewes et al., 2003) which greatly affects consumer acceptance. According to their respective positions in the scatter plot the breeding selections UCI 19-60 and UCI 19-19 may be categorized as medium and low phenolic content genotypes. The breeding selection UCI 19-79 had slightly higher phenolic content than UCI 5-65 but lower than “Arbequina” cultivar. The localization of this selection in the center of the fourth quadrant (Figure 5B) matches that of luteolin and 3,4-DHPEA acetate in the vector distribution of the variables (Figure 5A). These two compounds are the most significant phenolic components in the VOO of UCI 19-79, which possesses the highest content of luteolin and 3,4-DHPEA acetate among all the genotypes analyzed (14.4 and 27,6 μg/g oil, respectively).

## Conclusion

The high correlation found between fruit and oil phenolic components content, as well as the high genotypic variance for them, indicate that the analysis of fruit phenolic compounds, without the previous step of oil extraction, is an useful tool for olive breeding which could facilitate the selection of olive genotypes with potential interest in terms of oil phenolic composition. In this sense, fruit phenolic profiling could be used as selection criterion in the early stages of olive breeding programs to avoid the selection of genotypes whose oils will never reach an optimum phenolic content (European Commission, 2012). Moreover, the low genotype × environment interaction on phenolic composition, leading to a similar ranking of genotypes on the different environments, could also facilitate the evaluation of new selections from breeding works. The analytical methodology reported in this study allowed the identification of the new selection UCI2-68, characterized by an optimum phenolic profile, together to a good agronomic performance previously reported (León et al., 2004a,b, 2007, 2011), which represents a new olive cultivar producing superior quality oil.

## Author contributions

RdlR and LL: Conceived and designed the breeding experiments; AP and CS: Designed and performed the analytical studies; AP: Wrote the manuscript. All authors discussed and commented the manuscript.

### Conflict of interest statement

The authors declare that the research was conducted in the absence of any commercial or financial relationships that could be construed as a potential conflict of interest.

## Footnotes

^1^http://faostat.fao.org/site/567/DesktopDefault.aspx?PageID=567#ancor
